# Draft Genome Sequences of Acinetobacter baumannii Isolates Recovered from Sewage Water from a Poultry Slaughterhouse in Germany

**DOI:** 10.1128/MRA.00553-19

**Published:** 2019-07-11

**Authors:** Mykhailo Savin, Marijo Parcina, Silvia Schmoger, Judith Kreyenschmidt, Annemarie Käsbohrer, Jens A. Hammerl

**Affiliations:** a Faculty of Agriculture, Institute of Animal Sciences, University of Bonn, Bonn, Germany; b University Hospital, Institute of Medical Microbiology, Immunology and Parasitology, University of Bonn, Bonn, Germany; c Department for Biological Safety, German Federal Institute for Risk Assessment, Berlin, Germany; d Institute for Veterinary Public Health, University of Veterinary Medicine, Vienna, Austria; University of Maryland School of Medicine

## Abstract

Acinetobacter baumannii is an important human pathogen usually associated with severe hospital-acquired infections. Here, we announce the draft genome sequences of two livestock-associated isolates recovered from sewage water from a poultry slaughterhouse in Germany. Short-read whole-genome sequencing was conducted to determine the genetic basis of their antimicrobial resistance phenotype.

## ANNOUNCEMENT

Acinetobacter baumannii strains belong to the most critical pathogens for health care institutions, as they can efficiently incorporate antimicrobial resistance from the environment or other bacteria in their genomes (1, 2). Bacteria of this species are associated mainly with hospital-acquired pneumonia and sometimes with infections of the central nervous system, skin, or soft tissue (1, 3). Acinetobacter bacteria are Gram-negative, oxidase-negative, nonmotile, nonfermenting coccobacilli that are ubiquitously distributed in nearly all environmental habitats (i.e., water and soil) (2, 4). To assess the impact of livestock-associated A. baumannii strains on human health, selected isolates from sewage water from a poultry slaughterhouse were further characterized phenotypically and genotypically.

Two isolates exhibiting different colony morphologies (LWGS-03-02-11A and LWGS-03-02-11B) were obtained from process water of eviscerators from a German poultry slaughterhouse in 2018 by plating sample material on CHROMagar extended-spectrum beta-lactamase (ESBL) medium (Mast Diagnostica, Reinfeld, Germany). After incubation at 42°C for 24 h, cream-opaque Acinetobacter-like colonies were streaked onto 5% sheep blood agar (Mast Diagnostica) and confirmed by oxidase testing. Both isolates were assigned to the Acinetobacter calcoaceticus-A. baumannii complex using matrix-assisted laser desorption ionization–time of flight mass spectrometry (MALDI-TOF MS) employing a Vitek MS system (bioMérieux, Marcy l’Etoile, France). Antimicrobial resistance testing was conducted by broth microdilution using Mueller-Hinton broth, according to the recommendations of the CLSI guidelines, as previously described (5). Despite their different colony morphologies and XbaI pulsed-field gel electrophoresis (XbaI-PFGE) profiles, LWGS-03-02-11A and LWGS-03-02-11B exhibited identical MIC values, as shown in Table 1. To characterize the genetic basis of both isolates, genomic DNA (gDNA) was extracted from liquid cultures grown in lysogeny broth (LB) using the PureLink genomic DNA minikit (Invitrogen, Carlsbad, CA, USA). DNA libraries for whole-genome sequencing (WGS) were prepared using the Nextera XT DNA sample preparation kit. Short-read sequencing (MiSeq reagent v3 600-cycle kit) was conducted on a MiSeq benchtop sequencer (Illumina, San Diego, CA, USA), as previously reported (5). Raw reads were provided as quality-trimmed sequences and were further checked and verified by FastQC (version 1.0.4; https://www.bioinformatics.babraham.ac.uk/projects/fastqc/). SPAdes *de novo* assemblies were conducted using PATRIC (version 3.5.21), while genome annotation was performed using PGAP of the NCBI database (6, 7). Default parameters were used for all software tools.

**TABLE 1:** Genetic features of Acinetobacter baumannii isolates LWGS-03-02-11A and LWGS-03-02-11B

Feature	Data for A. baumannii isolate^*a*^:	
	LWGS-03-02-11A	LWGS-03-02-11B
Parameters		
No. of reads (total)	2,284,028	2,911,344
Avg read length (bp)	147.73	147.83
No. of contigs	90	103
*N*_50_ (bp)	159,782	159,782
*L*_50_	8	8
Genome coverage (×)	>35	>45
Genome		
Size (bp)	3,983,337	3,982,846
GC content (%)	38.90	38.89
Genetic elements^*b*^ (no.)		
Total genes	3,925	3,852
Total CDS	3,854	3,781
Coding genes	3,744	3,672
Coding CDS	3,744	3,672
RNA genes	71	71
rRNAs (5S, 16S, 23S)	1, 1, 1	1, 1, 1
tRNAs	64	64
ncRNAs	4	4
Pseudogenes (no.)		
Total	110	109
Ambiguous residues	0	0
Frameshifted	41	40
Incomplete	60	60
Internal stop	26	26
Multiple problems	15	15
MLST^*c*^		
Abaumanni1	ST-836	ST-836
Abaumanni2	ST-388	ST-388
Database accession no.		
GenBank no.	RCUZ00000000	RCVA00000000
BioProject no.	PRJNA496252	PRJNA496253
BioSample no.	SAMN10237498	SAMN10237499
Genetic resistance determinants^*c*^		
Beta-lactams (%)	*bla*_OXA-71_ (99.88 [825/825]), *bla*_ADC-25_ (97.74 [1,152/1,152])	*bla*_OXA-71_ (99.88 [825/825]), *bla*_ADC-25_ (97.74 [1,152/1,152])
Phenotypic resistance (MIC, mg/liter)^*d*^		
Ampicillin	8	8
Azithromycin	≤2	≤2
Cefepime	1	1
Chloramphenicol	32	32
Ciprofloxacin	0.06	0.06
Colistin	≤1	≤1
Ertapenem	1	1
Cefotaxime	8	8
Cefoxitin	64	64
Gentamicin	1	1
Imipenem	0.25	0.25
Meropenem	0.25	0.25
Nalidixic acid	≤4	≤4
Sulfamethoxazole	≤8	≤8
Ceftazidime	2	2
Temocillin	>128	>128
Tetracycline	≤2	≤2
Tigecycline	≤0.25	≤0.25
Trimethoprim	16	16

a Both isolates were obtained in 2018 from sewage water in Germany.

b CDS, coding sequences; ncRNAs, noncoding RNAs.

c *In silico* analysis was conducted using the Web-based tool MLST finder 2.0 (software version 2.0.1) (for MLST data) and ResFinder 3.1 (software version 3.1.0) (for genetic resistance determinants) of the Center for Genomic Epidemiology (http://www.genomicepidemiology.org/). The percentages of nucleotide identity of the target sequence to the reference are given in parentheses, and the number of nucleotides covered by the identified resistance gene and that of the respective reference gene are given in brackets.

d Resistance testing was conducted according to the guidelines of the CLSI.

An overview of the genetic features and antimicrobial resistance profiles of both isolates is given in Table 1. The draft genomes of LWGS-03-02-11A and LWGS-03-02-11B exhibited little variability in their sizes (∼3.983 Mbp), G+C contents (38.8 to 38.9%), and numbers of different genetic elements. Furthermore, the two isolates belong to the same sequence type (ST), ST-836 (Abaumanni1) and ST-388 (Abaumanni2), using the two available multilocus sequence typing (MLST) schemes for A. baumannii typing of MLST finder 2.0 (software version 2.0.1) (8). Bioinformatics analysis using ResFinder 3.1 (software version 3.1.0) (9) revealed that both genomes harbor *bla*_OXA-71_ (99.88% nucleotide identity to accession number AY750913) and *bla*_ADC-25_ (97.74% nucleotide identity to accession number EF016355) coding for beta-lactam antibiotics.

To assess the impact of livestock-associated A. baumannii isolates on human health, comprehensive data on their antimicrobial resistance development and their genetic basis will be needed. However, until now, Acinetobacter species of livestock and food origins have not usually been monitored.

### Data availability.

The draft genome sequences of LWGS-03-02-11A and LWGS-03-02-11B were deposited in GenBank under accession numbers RCUZ00000000 (BioProject number PRJNA496252) and RCVA00000000 (BioProject number PRJNA496253), respectively.
